# Retraction Note: Glycyrrhizic acid ameliorates submandibular gland oxidative stress, autophagy and vascular dysfunction in rat model of type 1 diabetes

**DOI:** 10.1038/s41598-023-49826-3

**Published:** 2024-01-03

**Authors:** Saad Mohamed Asseri, Nehal M. Elsherbiny, Mohamed El-Sherbiny, Iman O. Sherif, Alsamman M. Alsamman, Nadia M. Maysarah, Amira M. Elsherbini

**Affiliations:** 1 Department of Clinical Medical Sciences, College of Medicine, AlMaarefa University, P.O. Box 71666, Riyadh, Saudi Arabia; 2 Department of Biochemistry, Faculty of Pharmacy, Mansoura University, Mansoura, 35516 Egypt; 3 Department of Pharmaceutical Chemistry, Faculty of Pharmacy, University of Tabuk, 71491 Tabuk, Saudi Arabia; 4 Department of Basic Medical Sciences, College of Medicine, AlMaarefa University, P.O. Box 71666, 11597 Riyadh, Saudi Arabia; 5 Department of Anatomy, Faculty of Medicine, Mansoura University, Mansoura, Egypt; 6 Faculty of Medicine, Emergency Hospital, Mansoura University, Mansoura, 35516 Egypt; 7 African Genome Center, Mohammed VI Polytechnic University, Ben Guerir, Morocco; 8 Agricultural Genetic Engineering Research Institute, PO Box 12619, Giza, Egypt; 9 Department of Pharmacology and Toxicology, College of Pharmacy, Qassim University, Buraydah, Saudi Arabia; 10 Department of Oral Biology, Faculty of Dentistry, Mansoura University, Mansoura, 35516 Egypt

Retraction of: *Scientific Reports* 10.1038/s41598-021-04594-w, published online 14 January 2022

The Editors have retracted this Article.

After publication, concerns were raised regarding repetitive features in the images presented in Fig. 4C, 5A, B and D. The Authors have shared the raw data for validation; however, the Publisher has found notable differences between the raw data and published images. The Editors therefore no longer have confidence in the presented data.

Nehal M. Elsherbiny and Amira M. Elsherbini do not agree to this retraction. Saad Mohamed Asseri, Iman O. Sherif and Nadia M. Maysarahnot have not responded to any correspondence from the editor or publisher about this retraction. The publisher has not been able to obtain current email addresses for Mohamed El-Sherbiny and Alsamman M. Alsamman.

